# When There Is Pain After Hernia Surgery - Patient Perspectives

**DOI:** 10.3389/jaws.2024.13683

**Published:** 2024-10-24

**Authors:** J. Bullock, S. Blackwell, E. Keus, M. R. M. Scheltinga, W. A. R. Zwaans

**Affiliations:** ^1^ Patient Advisory Committee European Hernia Society, Blackburn, United Kingdom; ^2^ Hernia Patient Representative, Liverpool, United Kingdom; ^3^ Chronic Postoperative Inguinal Pain Patient Representative, Apeldoorn, Netherlands; ^4^ Department of General Surgery, Máxima Medical Centre, Veldhoven, Netherlands; ^5^ SolviMáx, Centre of Excellence for Abdominal Wall and Groin Pain, Eindhoven, Netherlands; ^6^ NUTRIM School of Nutrition and Translational Research in Metabolism, Maastricht University Medical Centre, Maastricht, Netherlands

**Keywords:** chronic pain, pain, postoperative, chronic postoperative inguinal pain, hernia, herniorrhaphy, patient-centered care, patient outcome assessment

Dear Editors,

There are over 20 million hernia repairs carried out worldwide every year, with the incidence of chronic pain after inguinal hernia surgery (**CPIP**) thought to be up to 11% [1]. CPIP is defined as an unpleasant sensory experience in the groin which persists for more than 3 months following inguinal hernia repair. Biological, psychological and social factors all contribute to CPIP. This article focuses on the patient’s perspective regarding CPIP.

During an outpatient appointment a patient may report symptoms including a constant intense pain which often increases after activity. This pain can be sharp, it can be burning and it can travel down through the groin or radiate around the back. CPIP is relentless and can lead to a never ending vicious circle of pain-depression-anxiety-depression-pain. The patient may state that they are unable to sleep due to this pain yet they are still expected to work, to function as a partner, a husband, a wife, a lover, a parent or a friend. Sex is an important part of a relationship but CPIP does often not allow for intimacy and sadly many patients find that their relationships suffer and for some they may break down. Can you imagine the guilt patients with CPIP experience as parents, many feel as though they are letting their children down, they cannot run around and play soccer or take the children to the park because of the constant unrelenting pain.

Hernia surgeons acknowledge that the pathophysiology of CPIP is complex and to date there are no gold standard tests to diagnose CPIP. Additional imaging is most likely to be normal and the number of high quality evidence or strong based recommendations for treating CPIP are limited [1]. Is it not now time for the international hernia societies to prepare universally accepted recommendations for treating CPIP? McMasters University states that patients should be involved in the development of clinical guidelines and should be considered as experts in their condition because they have actual lived experience. By working together with CPIP patients on this an algorithm will surely benefit both patients and surgeons. Open access to accepted algorithms and pathways for both patients and surgeons will increase transparency.

In recent years there have been campaigns on Twitter and Facebook questioning the use of surgical mesh and whilst these originated from the pelvic mesh patients many CPIP patients have since joined in this campaign. Frequent statements/postings include “when will surgeons start to listen to us, we are the ones who know our bodies, we can tell you when something does not feel right.” These campaigners will question if clinicians who follow CPIP literature actually listen to their patients without prejudgement. They believe there is a culture of gaslighting centred around chronic pain; indeed three of the authors of this contribution have both personally experienced not being listened to by their surgeon and felt they were dismissed. They felt let down and failed by the very people who were trusted to make them better. Today patients expect to play an active part in their treatment, they expect quality information to enable them to understand what is happening to their bodies, they want and need to be involved in any decision making regarding their treatment. The consequences of not being believed and not being listened to are far reaching as patients will find it difficult to trust any future clinicians, any consultations will be difficult. It is important that doctors are aware that how they communicate to patient matters.

Patients can become angry because the operation which was supposed to give them back their quality of life has in fact made things worse. Sadly not all surgeons understand CPIP and patients can find themselves being dismissed when all physical tests bloods, scans, etc., come back normal, yet to the patient the pain is real. The increased stress and anxiety from being dismissed and gaslighted can lead to an increase in a patients pain levels [2]. If a surgeon does not understand their pain how can patients expect others to? Sadly some patients have found themselves facing disciplinary action at work as employers are not empathetic to CPIP and to employees who have to repeatedly take time away from work or who physically cannot perform tasks.

The aspect of providing the patient with sufficient information during the informed consent process prior to surgery is often neglected. When a surgeon determines that a hernia repair using surgical mesh is required - as recommended by the (inter)national guidelines [1] - all patients should receive information on the procedure, associated risks and potential long term adverse sequelae. To date the majority of informed consent procedures fail to do so. It is a surgeons responsibility to fully inform patients, to provide information in either written or digital form and to offer patients the opportunity to ask questions. The responsibility of achieving a benign outcome is then shared by both parties once consent to treatment is granted by the patient.

In 2020 an analysis of mesh implant posts on common social media found that approximately 95% of the messages had a negative sentiment and that any prospective hernia patients who conducted their own internet research developed this negative perception of mesh implants [3]. Law firms have also used mesh related discussions and will encourage patients with any symptoms (related or unrelated) to file lawsuits, using the lack of informed consent as a basis for liability. These lawsuits are not necessarily beneficial to patients but they are likely to be highly beneficial to the law firms finances, contributing to their earnings. Claims are skyrocketing, especially in the United States [4] and are now spreading across Europe. In 2022 a study assessed the expectations of patients who started such a process for implant related complaints involving the newly formed Medical Disputes Committees in the Netherlands [5]. Interestingly the majority of patients did not initiate these complaints for financial compensation, their main goal was to be heard and receive treatment.

It is difficult to find social media sites whose content is both positive and factual. In February 2022 following discussion with the European Hernia Society (EHS) a Facebook group *Hernia Patient Support Group* was formed. This new group was a forum for patients to ask questions, to gain factual information and to support each other. Advice on how to cope with everyday life with hernia is provided. This group was created jointly by a chronic pain patient and dedicated hernia surgeons. It quickly grew in popularity showing that patients had been waiting for such a support group; to date there are over 5,200 members. By checking on posted messages by involved, well-informed patients and surgeons misinformation is avoided, inappropriate or resentful messages are deleted and professional opinions or advise is shared.

This support group has shown that patients want to trust their surgeons regarding mesh implants but there is still widespread concern over their use in hernia surgery. As surgeons you must understand this and take the time needed to reassure your patients. It is a surgeon’s duty and responsibility to properly inform patients about the recovery process and take away the anxieties about hernia surgery to repair trust in the procedure. By doing so, the responsibility of achieving a benign outcome is then shared by both parties.

In conclusion the authors of this letter would hope that surgeons will understand patient concerns and that future research will be conducted into the complexities of chronic pain and how best to treat this but for now compassion and understanding must be shown to those who suffer. No surgeon should add to a patients anxiety over chronic pain, all patients would agree that they would accept being told that their current surgeon could not help them so long as they were then referred to someone who was experienced in chronic pain. For chronic pain patients they may never get back their full quality of life but with your help they can often recover enough to allow them to enjoy life again.

## Data Availability

The original contributions presented in the study are included in the article/supplementary material, further inquiries can be directed to the corresponding author.
